# Importance of managerial roles in physician leaders’ work evaluated by medical students: a mixed methods approach

**DOI:** 10.1108/JHOM-02-2024-0034

**Published:** 2025-04-04

**Authors:** Sari Huikko-Tarvainen, Timo Tuovinen, Petri Kulmala

**Affiliations:** Faculty of Medicine, Oulu University, Oulu, Finland; Research Unit of Health Sciences and Technology, University of Oulu, Oulu, Finland; Medical Research Center Oulu, Oulu University Hospital, Oulu, Finland; Faculty of Science, University of Oulu, Oulu, Finland

**Keywords:** Medical student, Physician leader, Leadership, Management, Mintzberg

## Abstract

**Purpose:**

This study aims to investigate final-year medical students’ perceptions regarding the significance of different managerial roles in fulfilling physician leaders’ work.

**Design/methodology/approach:**

In 2020, an electronic questionnaire was distributed to all final-year medical students at the University of Oulu, Finland. A quantitative analysis of managerial roles, based on Mintzberg’s model, utilized statements rated on a five-point Likert scale. Statistical assessments examined differences in role importance relative to students’ age, gender, and educational background. The free-text responses underwent qualitative deductive content analysis and quantification.

**Findings:**

In total, 110 participants (68%) responded. Both quantitative and qualitative analyses underscored the high importance of different managerial roles in leadership work. Female respondents attributed greater importance to the monitoring role (mean Likert scale score of 4.6 vs. 4.1, *p* < 0.0001). Age exhibited a slight negative correlation with the disseminator role (rs = −0.2, *p* = 0.04), while previous higher education did not significantly influence the importance of Mintzberg’s roles.

**Practical implications:**

This study underscores the necessity for physicians to embody different managerial roles, emphasizing the need for comprehensive leadership education to manage operations and lead subordinates. The heightened importance assigned to the monitoring role by female students is significant, considering the increasing number of female physicians. Mintzberg’s framework could offer an additional tool for leadership education in medical curricula.

**Originality/value:**

This study marks the first exploration of final-year medical students’ perspectives on the importance of managerial roles in the physician leaders’ work through Mintzberg’s model.

## Introduction

### The essentiality of leadership skills

To improve healthcare system performance, leadership skills have been identified as essential for future physicians (Lindgren and Gordon, 2016) and, consequently, for medical education. Clinical leadership is linked to better organizational and patient outcomes (Sarto and Veronesi, 2016; Tasi *et al.*, 2019). Furthermore, societies expect leadership skills from physicians (Angood and Shannon, 2014), and most physicians prefer that their leaders emerge from within their own profession (Berghout *et al.*, 2017; Clay-Williams *et al.*, 2017; Huikko-Tarvainen, 2022; Perry *et al.*, 2017). Additionally, the circumstances of leadership in healthcare are evolving over time (Koskiniemi *et al.*, 2019). For example, the educational background of medical students has advanced beyond high school (Aston-Mourney *et al.*, 2022), and there is an increasing number of female physicians worldwide (Finnish Medical Association, 2019; Kusurkar *et al.*, 2010). Moreover, leadership is an evolving concept (Silva, 2016) and is not universal, but may differ based on gender or culture (Ayman and Korabik, 2010).

Physician leaders from earlier generations often gained leadership positions through clinical or research achievements, with less emphasis given to leadership and management skills, experience, or education (Huikko-Tarvainen *et al.*, 2021; Perry *et al.*, 2017). However, it has been argued that medical education alone does not necessarily lead to formal leadership competence, although it provides a solid basis for understanding healthcare services and gaining the trust of the physicians whom they lead (Finnish Medical Association, 2013). Furthermore, several literature reviews and studies have emphasized the need to improve specific leadership and management skills among physicians at various career stages or on various paths (Angood and Shannon, 2014; Geerts *et al.*, 2020; Huikko-Tarvainen *et al.*, 2021). Physician leaders have also identified the necessity of strengthening their professional leadership identities (Huikko-Tarvainen *et al.*, 2021; Quinn and Perelli, 2016; Spehar *et al.*, 2015).

Additionally, the COVID-19 crisis altered healthcare circumstances such that not only physician leaders, but also frontline physicians, must offer support to formal leaders in making important public health decisions. Consequently, they need to demonstrate leadership and management skills, not only as part of a gradual progression to formal leadership positions but also as part of their daily work (Rotenstein *et al.*, 2021). Although the development of a leader’s identity is a continuous process (Koskiniemi *et al.*, 2019) and physicians’ leadership identity also strengthens over their working years (Danish Leadership and Management Commission, 2017–2018., 2018; Ministry of Social Affairs and Health, 2020), this pace of development is currently insufficient. As the foundation of a physician’s professional identity formation starts during medical school and can be further developed by pursuing additional training (Chan *et al.*, 2018), it is essential to understand how to train and adopt leader’s identity and support the development of leadership skills (Quinn and Cola, 2020).

Since medical students are involved in the activities of physicians under certain circumstances in healthcare (Valvira, 2021), this also partly applies to them. Furthermore, as perceptions tend to direct actions, motivation, and behavior (Koskiniemi *et al.*, 2019) in the present and future, the perceptions of medical students regarding leadership and management should be considered and researched for leadership education in medical curricula. However, research focusing on medical students’ perceptions of leadership and management roles in leadership education is scarce (Quince *et al.*, 2014), considering their roles as future physicians and potential physician leaders.

### Physician leadership education

The primary aim of physician leadership development programs has predominantly been to enhance physicians’ leadership capabilities and enhance organizational performance (Frich *et al.*, 2014). Two recent literature reviews of physician leadership development have concluded that leadership development programs can also result in enhanced outcomes at the individual, organizational, and patient levels (Geerts *et al.*, 2020; Lyons *et al.*, 2020). Moreover, the integration of faculty-mentored but student-led groups as a core curriculum intervention, providing undergraduate medical students with opportunities to enrich leadership experience alongside initiatives for patient safety and quality improvement, can bolster medical students’ readiness and confidence in assuming leadership roles in their future careers, effecting change in healthcare, and attaining leadership positions (Inayat *et al.*, 2023; Rotenstein *et al.*, 2019).

According to previous literature, medical students value “character,” “competence,” and “commitment” in leadership, along with exposure to examples of physician leadership and opportunities to engage in leadership activities. Additionally, they express a desire to participate in reflection exercises, which are recommended to enhance leadership development within medical curricula. (Vo *et al.*, 2023) Furthermore, a systematic review identified several domains as fundamental to medical leadership and management abilities in undergraduate medical education. These include quality improvement, managed care, resource utilization and healthcare costs, the doctor’s role, patient safety, and basic leadership and management principles (Abbas *et al.*, 2011). These findings align with the perspectives of experienced physicians and physician leaders (Huikko-Tarvainen, 2022).

Moreover, a recent comprehensive managerial framework derived from a review of the academic literature underscores that healthcare leadership skills should encompass a broad spectrum of competencies across ten main categories. These categories include specific competencies fundamental for effective healthcare management: “character, interpersonal relations, leadership, professionalism, soft human resource management (HRM), management, organizational knowledge, technology, knowledge of the healthcare environment, and change and innovation.” Understanding the external influences on healthcare organizations, including political, social, economic, and technological factors, as well as stakeholder roles and interactions, is essential for effective decision-making and strategic planning. Competencies related to leading change initiatives, anticipating resource needs, initiating improvements, and fostering innovation are essential for adapting to the evolving dynamics of healthcare landscapes and driving organizational progress. (Oskarsson and Vik, 2024)

### Challenges in leadership education in medical curricula

There is a consensus that leadership training should commence during undergraduate medical education (James *et al.*, 2021). However, only a minority of medical schools globally have formally incorporated courses in leadership into their curricula (Ireri *et al.*, 2011; Wagenschutz *et al.*, 2019). Conversely, in certain countries such as Finland, the defined outcomes and competencies for graduating physicians include leadership skills and an understanding of the physician’s role in healthcare service leadership and administration (University of Helsinki, 2020). Nevertheless, several challenges have impeded leadership learning and education within medical curricula. These include inadequate support for practicing leadership skills in hospital environments, a shortage of funds for organizing leadership education, insufficient time to integrate leadership education into the medical curriculum (James *et al.*, 2021; Matthews *et al.*, 2018; Omar *et al.*, 2020; Richard *et al.*, 2019), an absence of standardization in the leadership curriculum (Vo *et al.*, 2023), and a lack of awareness regarding the importance of physicians’ leadership competence and competing competencies to be learned in medical education (Omar *et al.*, 2020). Furthermore, a recent review of leadership development in postgraduate medical education reveals that interventions for developing leadership education lack grounded conceptual leadership frameworks, yield limited evaluation outcomes, and primarily concentrate on cognitive leadership domains. Consequently, leadership education should offer a conceptual framework and pursue a comprehensive approach (Matsas *et al.*, 2022; Sultan *et al.*, 2019; Vo *et al.*, 2023).

Thus, this study explores final-year medical students’ perceptions regarding the significance of various managerial roles in fulfilling a physician leader’s responsibilities through a mixed methods approach, incorporating Mintzberg’s framework theory (Mintzberg, 1980), a methodology rarely employed in the healthcare context.

## Theoretical framework

### Leadership and management

Leadership is a phenomenon comprising a process wherein a leader consciously influences other individuals by guiding, structuring, and facilitating activities and relationships within a group or organization to achieve a common goal (Northouse, 2016; Yukl, 2013). In the medical domain, this collective pursuit typically focuses on enhancing patient outcomes (Schmidt and Linenberger, 2020). The leadership process involves various means of exerting influence, both direct and indirect, and may involve one or both (Antonakis and Day, 2018, p. 5; Yukl, 2013, pp. 23, 36). A leader’s responsibilities usually encompass both leadership and management tasks (Yukl, 2013). The aim of management is to achieve efficiency, predictability, and order, while that of leadership is to effect change; the significance of each depends on the specific case (Kotter, 1990; Yukl, 2013). Moreover, some scholars posit that while the essence of management lies in achieving efficiency, leadership primarily concerns achieving effectiveness. Additionally, management focuses on methodology and execution (“how”), while leadership explores the purpose and rationale (“what” and “why”). Management involves establishing and maintaining systems, controls, procedures, policies, and organizational structure. In contrast, leadership focuses on fostering trust and understanding among individuals. It is characterized by innovation and the impetus to initiate change. While management often entails maintaining the existing state (“status quo”), leadership is marked by creativity, adaptability, and agility. Unlike management’s focus on immediate outcomes, leadership adopts a broader perspective, considering future possibilities beyond merely the bottom line. (Bennis and Goldsmith, 1997)

For decades, the leadership literature has engaged in an ongoing discussion not only regarding the distinctions between leadership and management but also concerning their interplay, respective significance, and potential for individuals to excel in both roles simultaneously (Yukl, 2013). For instance, Bennis and Goldsmith (1997, p. 4) posited that whereas “a good manager does things right, a good leader does the right things.” Some scholars have asserted that it is impossible to fulfill the roles of both a leader and a manager simultaneously (Yukl, 2013). Similarly, a study involving resident doctors revealed a clear dichotomy in their perceptions of management versus leadership, which aligns with another study wherein junior doctors viewed management as less important than leadership (Barrow *et al.*, 2011). Furthermore, the terms “leader,” “manager,” and “boss” are frequently employed interchangeably to describe individuals occupying positions where they are tasked with exhibiting either leadership or management, or both (Yukl and Gardner, 2020, p. 27). Likewise, the work of a physician leader often entails a hybrid nature, encompassing leadership and management tasks as well as clinical patient work (Berghout *et al.*, 2017; Huikko-Tarvainen, 2022). Despite ongoing discourse, various leadership studies have emphasized the significance of leadership and management, elucidated their distinctions whilst acknowledged their intersections (Yukl, 2013, pp. 22–26). That is, leadership skills are complemented by management (Kotter, 1990, pp. 4–7). This perspective is corroborated by Mintzberg’s managerial role theory, which views leadership as one of the arrays of managerial functions (Mintzberg, 1980, pp. 54–99).

### Mintzberg’s model of the 10 managerial roles

Mintzberg proposed a model highlighting ten managerial roles (Mintzberg, 1980), among which leadership entails the motivation to lead and the creation of favorable working conditions. Although the remaining nine roles signify different kinds of managerial responsibilities, leadership plays a unique role that affects the other roles. Based on Mintzberg, managerial activities can be divided into three key categories: “interpersonal,” “informational,” and “decisional.” Specifically, interpersonal roles can be further subdivided into three sub-roles: the “figurehead,” “leader,” and “liaison” roles, each conferring on the manager a distinct position for acquiring information. In the figurehead role, the manager fulfills formal and symbolic obligations, symbolizing formal authority without involvement in information processing or decision-making. In the leader role, the manager provides direction, imbues the organization with purpose, and shapes its culture. As a liaison, the manager fosters horizontal relationships, connecting the organization with its external environment and gathering pertinent information. Similarly, informational roles can be divided into three sub-roles: the “monitor,” the “disseminator,” and the “spokesman.” In the monitor role, the manager seeks knowledge to comprehend organizational and environmental dynamics, ensuring the organization operates smoothly. As the disseminator, the manager transfers information from external sources into the organization and shares it with subordinates. In the spokesman role, the manager communicates organizational information to external stakeholders. Finally, decisional roles encompass four sub-roles: the “entrepreneur,” “disturbance handler,” “resource allocator,” and “negotiator.” In the entrepreneur role, the manager instigates and oversees organizational changes, serving as a catalyst for transformation. As a disturbance handler, the manager addresses conflicts and restores organizational equilibrium. In the “resource allocator” role, the manager prioritizes and distributes resources while making major decisions. Finally, in the negotiator role, the manager engages in negotiations on behalf of the organization (Mintzberg, 1980, pp. 56–91.)

### A research gap to fill

There is a noticeable dearth of empirical evidence and theoretical discourse concerning the perceptions and practices of physician leaders in fulfilling their managerial responsibilities. The literature strongly advocates for the adoption of managerial frameworks guided by physician leadership. (Ireri *et al.*, 2011) Consequently, there is a pressing need for leadership education in medical training to offer a conceptual framework and adopt a comprehensive approach; however, such a model remains conspicuously absent (Matsas *et al.*, 2022; Sultan *et al.*, 2019; Vo *et al.*, 2023).

To assess and interpret leadership education within medical curricula, gain deeper insights into leadership development during residency training, and effectively prepare future cohorts of physician leaders, particularly considering potential generational shifts in mindset, this paper presents a perspective utilizing a mixed methods approach, incorporating Mintzberg’s framework to examine final-year medical students’ perceptions of the significance of managerial roles (Mintzberg, 1980) in the fulfillment of physician leaders’ duties. Such an investigation represents a novel contribution to the literature and thus addresses a significant gap in leadership research.

## Data and methods

### Study design

To ensure the content validity of the study, the survey instrument underwent careful development, drawing upon a comprehensive review of the literature on physician leadership, combined with insights from medical students regarding physician leadership and its integration into medical curricula (Ruel and Ruel, 2019). Before full implementation, the survey was pretested by the authors to assess the reliability and clarity of the survey elements and to identify any possible ambiguities or difficulties in interpretation (Ruel and Ruel, 2019). Feedback obtained during this pretesting phase ensured that the questionnaire was both relevant to the study population and easy to understand, while maintaining alignment with the study objectives (Ruel and Ruel, 2019).

A survey methodology was chosen for this study because it efficiently collects data from large samples within a constrained timeframe. To enhance the reliability of the study, the survey was administered electronically and conducted in Finnish, using a web-based platform (Webropol) to ensure easy access and completion for participants. Additionally, the sample of sixth (final)-year students effectively represented the population of interest, as the entire cohort of final-year medical students from 2020 was invited to participate, resulting in a high response rate of 68% (110 out of 162).

Moreover, to establish content reliability and construct validity (Messick, 1995; Nha, 2021), a mixed methods approach was employed (Basham *et al.*, 2010; Messick, 1995; Nha, 2021). The formulation of the qualitative research question closely aligned with the research objectives, while the research design was further supported by using Mintzberg’s managerial framework and a 5-point Likert scale. Mintzberg’s managerial framework, a well-established theoretical model of managerial roles developed over the past 50 years, provided a robust conceptual basis for this study (Mintzberg, 1980). The quantitative questionnaire instrument was designed based on constructs from Mintzberg’s framework (Mintzberg, 1980), which has been validated in organizational studies. This approach strengthened the construct validity of the study (Ruel and Ruel, 2019). Likewise, Likert-based instruments have demonstrated strong construct validity and internal consistency in prior applications and are commonly used in medical education studies (Artino *et al.*, 2018; Sullivan and Artino, 2013). Thus, content evidence was supported by leveraging established instruments, ensuring that the questionnaire’s content accurately reflected its intended measurement goals (Ruel and Ruel, 2019).

As with every research instrument, Mintzberg’s framework has limitations, such as its limited ability to identify similarities due to its focus on differences, as well as challenges related to contextual relevance and validity across various cultural settings (Ansoff, 1991; Mount and Bartlett, 1999; Pearson and Chatterjee, 2003; Shapira *et al.*, 1980). Yet, it has proven valuable for studying managerial roles and has obtained reliable results in previous studies (Arman *et al.*, 2009; Glick, 2011; Guo, 2002; Mount and Bartlett, 1999; Pearson and Chatterjee, 2003; Rechtien *et al.*, 2022). These findings further support the reliability and validity of the research instrument (Ruel and Ruel, 2019). Additionally, the literature supports the use of Mintzberg’s framework in research on leaders’ managerial roles (Glick, 2011; Mount and Bartlett, 1999), including its application in healthcare contexts (Arman *et al.*, 2009; Guo, 2002; Pearson and Chatterjee, 2003; Rechtien *et al.*, 2022).

Hence, due to the comprehensive use of Mintzberg’s framework in managerial role studies, and its underutilization in exploring final-year medical students’ perceptions of the managerial roles required for physician leaders, the classification of Mintzberg’s roles was deemed appropriate as a quantitative research instrument in this study.

### Data analyses

**Figure 1:**
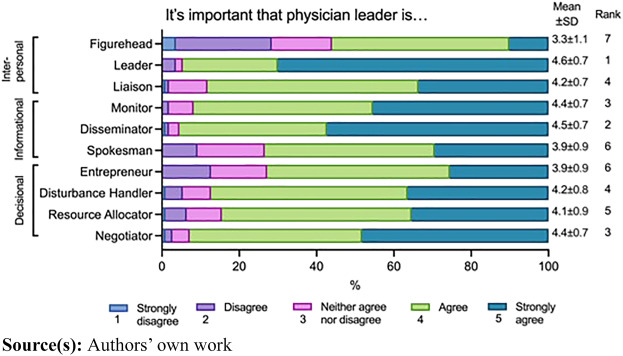
Mintzberg’s managerial roles (Mintzberg, 1980) in physician leaders’ work, as evaluated by medical students on a 5-point Likert scale. The ranking indicated on the right refers to Likert scale values varying from most (1) to least (7) important

A mixed methods approach was adopted to enhance the robustness of the study, considering the amount of data (*n* = 110) drawn from the quantitative parts of the study. The quantitative analysis established a descriptive framework, serving as a basis for comparison with the findings from the qualitative aspect of the investigation. Initially, guided by Mintzberg’s framework (Mintzberg, 1980), the study quantitatively evaluated importance of managerial roles (including “figurehead,” “liaison,” “leader,” “monitor,” “disseminator,” “spokesman,” “entrepreneur,” “disturbance handler,” “resource allocator,” and “negotiator”) in physician leaders’ work using a 5-point Likert scale (1: Strongly disagree, 2: Disagree, 3: Neither agree nor disagree, 4: Agree, 5: Strongly agree) (Figure 1).

Given the presumed familiarity of final-year medical students with physician leadership and management concepts, stemming from their exposure to leadership studies since the inception of their medical curriculum, it was assumed that they possessed a foundational understanding. To ensure clarity, Mintzberg’s managerial roles and their respective definitions were elucidated within the electronic questionnaire. Furthermore, the study investigated correlations between demographic variables (such as age, gender, and highest level of education prior to medical school) and the perceived significance of Mintzberg’s management roles. All the quantitative analyses were conducted using the IBM SPSS statistical package version 26.0 (IBM Corp., released 2013, Armonk, NY, USA). Gender disparities were assessed utilizing Student’s *t*-test, while correlations between assessments of Mintzberg’s roles on the Likert scale and the influence of age and previous education were examined through the Spearman correlation test, with two-sided *p*-values <0.05 deemed statistically significant.

Subsequently, a qualitative methodology was adopted (Eriksson and Kovalainen, 2008). Participants were presented with an open-ended question: “How should physicians be led?” This singular qualitative question was chosen to elicit a diverse array of responses that were unrestricted by narrow, specific topics. The responses, spanning four A4-sized pages (Arial font, 12-point, single spacing), were digitally stored and systematically coded to facilitate analysis. The data obtained from the open-ended question were not linked to the participants’ background variables.

In the subsequent phase, a content analysis was conducted employing a deductive approach, wherein the analytical framework was constructed based on existing knowledge (Eriksson and Kovalainen, 2008), which in this study pertains to Mintzberg’s framework. This process involved identifying Mintzberg’s emergent managerial roles from the qualitative data. Initially, a thorough comprehension of the data was achieved through iterative readings. Fundamental codes (words and phrases) representing the raw data were subsequently clustered, merged, and categorized. The managerial roles delineated by Mintzberg’s framework were identified from these categories. These roles were further refined and categorized into subcategories until distinct and consistent differentiations emerged. Subsequently, an analysis was conducted to ascertain how Mintzberg’s managerial roles were manifested in the qualitative data. Informative citations were then selected to exemplify researchers’ interpretations, with any identifying details removed or replaced to maintain anonymity. Such modifications did not materially impact the analysis or results but were undertaken solely to preserve anonymity. Additionally, a quantification analysis of the qualitative data (Schreier, 2012) was performed by calculating the occurrence rates of Mintzberg’s roles (*n*/%) within the dataset. Quantification provides a further angle to interpret the qualitative data (Grove *et al.*, 2012; Patton, 2015) and facilitates a more nuanced discussion (Halevi Hochwald *et al.*, 2023), and bolsters the study’s reliability and validity by triangulation (Eriksson and Kovalainen, 2008). Due to the limited volume of data within each subcategory of Mintzberg’s managerial roles, a more extensive statistical analysis of the qualitative data was not performed. Finally, the quantitative and qualitative findings were juxtaposed for comparison.

These analyses were independently conducted twice and subsequently reviewed by all authors to mitigate bias. Throughout the analysis and coding phases, regular meetings were held between the primary author and other contributors to gather feedback and refine the coding system. To improve the validity and reliability of the research findings, multiple types of triangulation were employed: (1) methodological triangulation, involving the integration of qualitative and quantitative data; (2) method triangulation, where various methods and analytical techniques were used to corroborate the results; and (3) researcher triangulation, with three researchers examining the data and cross-verifying their interpretations and conclusions (Eriksson and Kovalainen, 2008). Furthermore, maintaining methodological rigor further enhanced the validity and reliability of the results (Braun and Clarke, 2019).

### Ethical statement

Email invitations sent to final (sixth)-year medical students provided clear information detailing the study’s purpose, the voluntary nature of participation, guarantees of confidentiality and anonymity, and participants' right to withdraw or decline the use of their data at any time. Consent for data collection was obtained from all participants through the questionnaire, and permission for research usage was also secured. Data were analyzed without any personal identifiers, and no incentives were offered for participation. The study adhered to national and international research ethics standards for nonmedical research involving human participants, in accordance with the ethical guidelines of the Finnish National Board on Research Integrity TENK (2019) and the European Union’s data protection regulations. Approval for the study was granted by the Faculty of Medicine in compliance with its current policies.

## Results

### Characteristics of the informants

Of the total respondents, 53% (*n* = 58) were female, 46% (*n* = 51) were male, and one student did not indicate their gender. Most informants (*n* = 71/65%) fell within the 25–30 age bracket, while 14% (*n* = 16) were aged 31–35, 4% (*n* = 4) were aged 36–40, 8% (*n* = 9) were under 25, and 9% (*n* = 10) were over 41. Furthermore, 70% of respondents (*n* = 77) indicated that high school as their highest educational background before medical school, while 30% (*n* = 33) held a previous higher education degree.

### Results of the quantitative data

According to the data, all studied Mintzberg’s managerial roles were deemed important in physician leaders’ work; over 50% of the informants agreed that the roles were essential (Figure 1). However, significant differences were observed among the different roles. The most important role for a physician leader (rank 1) was that of a leader, with nearly 95% of the informants indicating its importance (mean Likert scale value of 4.6). Additionally, other highly ranked roles included those of a disseminator (96%, rank 2) and a monitor (92%, rank 3). Moreover, the role of the negotiator was also seen as significant (rank 3). However, contrary to the above results, 28.4% of the informants disagreed that a physician leader should serve as a figurehead.

The study also analyzed whether the informants’ background variables influenced the perceived importance of Mintzberg’s managerial roles. The female informants considered the monitoring role to be more important than the male participants did (mean Likert scale value 4.6 vs. 4.1, *p* < 0.0001). Additionally, age exhibited a slightly negative correlation with the disseminator role (rs = −0.2, *p* = 0.04), indicating that older informants attributed less importance to the physician leader’s role as a disseminator. No significant differences were observed between the other roles and background variables. Furthermore, having a higher education degree beyond high school did not significantly affect students’ perceptions of the importance of Mintzberg’s roles.

### Results of the qualitative data

In the qualitative segment of the study, all the roles specified in Mintzberg’s framework were evident in the data, and most of the participants (*n* = 110) provided responses that included several different managerial roles, generating a total of 186 words or phrases pertaining to the roles outlined in Mintzberg’s framework. According to the quantification of the qualitative data, the leader role exhibited the highest single occurrence (39%), while the remaining managerial roles collectively represented 61% (Table 1).

**Table 1:** Mintzberg’s model of managerial roles (Mintzberg, 1980), their occurrence rate (*n*/%) in the data (quantification) with the results of the content analysis and informative citations of the qualitative data

Managerial role activity	The sub-role and its appearance (*n*/%) in the data	Results of the content analysis	Citations
Interpersonal	Figurehead (7/3.8%)	The interaction of leadership and perceived status has a significant impact on organizational dynamics and interpersonal relationships. This principle suggests that the leader’s behavior acts as a catalyst for promoting compliance with established norms or guidelines and thus serves as a role model	Leading by example
			The so-called status of the leader must be maintained so that others listen to/follow
	Leader (73/39.2%)	A holistic view of physician leadership extends beyond technical proficiency to encompass effective leadership of interpersonal relationships, fostering trust, engaging in collaborative mentoring, and nurturing a supportive work environment. Physician leaders are responsible not only for clinical oversight but also for the development and well-being of their team	[Physician leaders should lead] like experts, meaning not only leading things, but also leading people
			By creating an atmosphere of trust that enables subordinates to learn and achieve common goals as well as care for subordinates’ well-being at work
			Guiding and mentoring subordinates so that they can complete their work
	Liaison (4/2.2%).	At the core of this role is the recognition that the success of leadership in healthcare organizations requires coordination and cooperation between professionals with diverse expertise. By promoting a shift towards interprofessional teamwork, this approach seeks to utilize the combined knowledge and interactions of various healthcare providers to advance a common goal	To foster awareness among various professional fields (e.g. nurses, physiotherapists, etc.) regarding the importance and implementation of cooperation between them
Informational	Monitor (27/14.5%)	This role involves not only identifying the clinical demands and responsibilities associated with medical roles but also understanding the contextual factors that influence the professional work environment. It highlights the importance of leadership sensitivity to the perspectives and demands of frontline medical professionals	By listening to doctors
			The leader should have a comprehensive understanding of the realities of a doctor’s work
	Disseminator (16/8.6%)	Central to this role is the recognition of the central role of proactive information dissemination, particularly in healthcare environments characterized by rapidly changing conditions, uncertainty and complexity. This strategy can increase flexibility and collective response to significant contemporary challenges	To disseminate information about significant current changes (e.g. corona information)
	Spokesman (7/3.8%)	A key aspect of this role is recognizing the value of delegating tasks and streamlining processes to improve efficiency and effectiveness in healthcare environments. The strategic reorganization of roles aims to maximise the utilization of the specialized knowledge and expertise of medical professionals	[Leaders] must try to eliminate the jobs of nurses, nurses, secretaries, and cleaners from doctors, allowing doctors to concentrate on their medical responsibilities
Decisional	Entrepreneur (13/6.9%)	This role emphasizes proactive planning and foresight in professional contexts, emphasizing the importance of proactive measures to address emerging needs and capitalize on developing opportunities. Central to this role is the recognition of the dynamic nature of professional environments, characterized by a combination of internal and external factors that shape organizational trajectories	To plan improvements for the future
			To consider the environment where we work, the opportunities, challenges, and development areas
	Disturbance handler (6/3.2%)	At the core of this role is the recognition of leadership as a dynamic and situational phenomenon, where contextual requirements necessitate adaptation of leadership style and behavior. In particularly challenging situations, physician leaders are expected to demonstrate strong leadership qualities such as flexibility, determination and confidence	In challenging situations, [leader] assume a more prominent leadership role
	Resource allocator (20/10.8%)	Central to this role is recognizing the reciprocal relationship between well-being at work and organizational performance, whereby providing adequate resources and support mechanisms improves productivity, satisfaction and employee retention. Equipping employees with the necessary skills, knowledge and resources to succeed in their roles not only increases individual job satisfaction and performance but also fosters a culture of continuous learning and improvement	Try to create reasonable working hours and tasks within the limits allowed by resources
			This includes, for example, organizing working conditions as well as possible, supporting subordinates at work and investing in their training and orientation
	Negotiator (13/6.9%)	This role emphasizes the importance of establishing common rules to guide coordinated action and ensure quality assurance while preserving the independence of physicians to use professional judgment and expertise. It emphasizes the importance of continuous development discussions and professional guidance as key mechanisms in promoting team cohesion and effective management	There should be established common rules for the workplace, but at the same time, allowing doctors to maintain autonomy in their decision-making processes
			Development discussions and professional guidance in the workplace are essential for fostering team spirit, which in turn makes leadership work easier

**Source(s):** Authors’ own work

## Discussion

The aim of the present study was to explore the perceptions of final-year medical students regarding the significance of various managerial roles in fulfilling the duties of physician leaders. The qualitative analysis of the study yielded a robust endorsement of the leadership role. Physicians anticipate that their leaders recognize their expertise, support their well-being, provide guidance and mentorship, foster a supportive work environment conducive to learning and task accomplishment, and coordinate efforts toward both personal and collective goals. These findings resonate with Mintzberg’s fundamental proposition concerning the function of a leader, which is to facilitate the alignment of individual aspirations with organizational objectives. (see Mintzberg, 1980, p. 62.) Furthermore, the findings of the current study underscore the importance of physician leaders understanding the unique needs of each physician, thus fostering a supportive and equitable environment. This notion also corresponds with Mintzberg’s portrayal of the leader’s role, wherein the manager shapes the organizational atmosphere. Consequently, through the leader role, the manager integrates diverse elements into a unified organization. (Mintzberg, 1980, pp. 60–62.)

Moreover, building upon the insights gleaned from the qualitative segment of the study, Mintzberg’s other managerial roles predominantly center on cultivating environments conducive to the independent professional endeavors of physicians. In the disseminator role, a physician leader possesses comprehensive knowledge across various healthcare disciplines, ensuring that physicians are kept informed of relevant changes. In the liaison role, the importance of interprofessional collaboration in healthcare is acknowledged. Recognizing the constraints imposed by available resources, the physician leader, acting as a resource allocator, endeavors to optimize working conditions and enable physicians to fulfill their duties within reasonable limits. In the spokesman role, the physician leader authorizes physicians to concentrate on their medical duties, while leading by example aligns with the expectations associated with the figurehead role. In the monitor role, the effectiveness of the physician leader is enhanced when the leader is intimately familiar with the realities of physicians’ work. Consequently, the leader seeks information by involving physicians in decision-making processes, thereby fostering a positive working community. In more challenging situations, where clear direction and constructive feedback are fundamental, the necessity of the disturbance handler role becomes evident. While standardized rules are essential, physicians should be granted autonomy in decision-making, a principle ensured by the negotiator. Furthermore, in the negotiator role, developmental discussions promote team cohesion, thereby facilitating the leader’s work. Lastly, in the entrepreneurial role, the leader specifies future development targets, along with their associated opportunities and challenges, all while bearing in mind the practicalities of physicians’ work. (Mintzberg, 1980, pp. 56–91.) Additionally, an equitable and individualized approach to each led physician, encompassing both leadership and managerial dimensions, was anticipated, aligning with prior literature emphasizing the importance of both leadership and management (Yukl, 2013, pp. 22–26).

Based on the quantitative segment of the study, the role of a leader (leadership) emerged as the most imperative individual role, consistent with the descriptions outlined in Mintzberg’s model, which is also congruent with the findings from the qualitative data. Moreover, all the managerial roles within Mintzberg’s framework were perceived as highly valuable in fulfilling the responsibilities of a physician leader. This was further evidenced by the free-text responses, wherein all managerial roles collectively garnered 61% support in the quantification of the qualitative data results. These outcomes regarding leadership and managerial functions are in line with recent studies involving more experienced physicians and physician leaders (Huikko-Tarvainen, 2022; Oskarsson and Vik, 2024), as well as research examining the definition of general leadership (Northouse, 2016; Yukl, 2013). Consequently, these findings echo the discourse suggesting that leadership is complemented by management roles (Kotter, 1990).

However, our findings diverge from previous studies wherein resident doctors maintained a distinct separation in their perceptions of management and leadership, with junior doctors regarding the former as inferior to the latter (Barrow *et al.*, 2011). This disparity may arise from differences in the stages of medical education (medical students versus second-year residents) and perceptions influenced by prior training and work experience.

Notably, our study was conducted amid the ongoing COVID-19 pandemic crisis, underscoring the imperative to foster both leadership and management skills. This aligns with the argument that the COVID-19 crisis has necessitated all physicians to assume managerial and leadership roles alongside their primary responsibilities as physicians (Rotenstein *et al.*, 2021). Furthermore, the significance of different managerial roles did not seem to depend on prior higher education before medical education, presenting novel insight from our research.

Another novel finding of the current study is that female medical students rated the monitoring role of leaders (seeking information to understand what is happening in the organization and its environment) as more significant than male participants. Among the participants, 53% (*n* = 58) were female, which aligns with the current landscape of leadership in healthcare (Koskiniemi *et al.*, 2019), wherein there is a rising number of female physicians worldwide (Finnish Medical Association, 2019; Kusurkar *et al.*, 2010). The discovery regarding the role of monitor may stem from the sample size of the quantitatively analyzed data or reflect previous findings suggesting that gender and culture can influence perceptions of leadership (Ayman and Korabik, 2010). However, despite these insights, women in academic medicine encounter slower career progress than men, who are also more likely to hold leadership roles and attain higher academic ranks (Carr *et al.*, 2018; Ringdahl *et al.*, 2014; Roth *et al.*, 2016). In contrast, given that leadership is an evolving concept (Silva, 2016) and that perceptions tend to shape actions, motivation, and behavior (Koskiniemi *et al.*, 2019), not only in the present but also in the future, these findings warrant careful consideration.

The third novel finding of the present study was that the older the participants were, the less significance they attributed to the physician leader performing the disseminator’s role, which entails distributing external information across the organization and internal information among subordinates. Generational differences may not entirely account for this trend, as the evolving identity of physician leaders tends to become more pronounced over the course of their careers (Ministry of Social Affairs and Health, 2020), although physician leaders from earlier generations were frequently promoted to leadership positions primarily on the basis of their clinical accomplishments, with less emphasis on their exposure to leadership and management education (Huikko-Tarvainen *et al.*, 2021; Perry *et al.*, 2017). Hence, the findings may be coincidental, given the sample size of the quantitatively analyzed data. This is further supported by the qualitative segment of the study, where the disseminator’s role was associated, for instance, with delivering vital information about the most significant current changes (e.g. COVID-19 information). This finding aligns with previous leadership studies that underscore the importance of communication skills in physician leadership (Huikko-Tarvainen, 2022; Oskarsson and Vik, 2024).

The previous literature provides various frameworks to elucidate the multifaceted nature of managerial roles and competencies essential for effective organizational leadership. The Mintzberg managerial framework (Mintzberg, 1980) utilized in this study bears noticeable resemblance to a recent holistic framework employed in the healthcare context (Oskarsson and Vik, 2024). Consequently, the current study offers insights into the applicability of Mintzberg’s framework for exploring managerial roles in the realm of physician leadership. Both frameworks converge on the recognition of leadership work as a complex endeavor necessitating a diverse set of roles and competencies, while emphasizing the pivotal role of interpersonal skills such as communication, teamwork, and relationship-building in managerial effectiveness. Additionally, both frameworks underscore the significance of the leader role, highlighting the manager’s ability to influence others, set direction, and inspire action toward organizational goals. However, these frameworks diverge in certain aspects. First, while Mintzberg’s framework provides a concise delineation of managerial roles within an organization, the holistic framework offers a more detailed breakdown of competencies across multiple dimensions, including character, professionalism, and knowledge transformation. As the holistic framework is contextualized within the healthcare domain, it also addresses competencies specific to healthcare management, such as patient-centered care, knowledge of the healthcare environment, and change and innovation in healthcare services. In contrast, Mintzberg’s framework is more generalizable across different industries and sectors. Additionally, Mintzberg’s framework does not explicitly address technology as a separate dimension of managerial competency. (Mintzberg, 1980; Oskarsson and Vik, 2024) In summary, the results analyzed through Mintzberg’s managerial framework suggest a spectrum of skills that can be categorized into personal and technical dimensions, as well as internal and external competencies within organizational contexts. These findings align with the results of a recent holistic broader framework of healthcare leadership competencies (Oskarsson and Vik, 2024). Thus, despite being conceptualized decades ago, the managerial roles defined by Mintzberg’s framework persist, rendering it applicable for evaluating diverse managerial roles in fulfilling the work of physician leaders in a concise manner.

## Conclusion

This mixed methods study highlights the indispensable and concurrent necessity for both leadership and managerial roles in physician leadership. Moreover, it addresses a gap in leadership research within the healthcare domain, providing nuanced knowledge of final-year medical students’ perspectives on leadership and managerial roles in physician leadership by also incorporating Mintzberg’s model (Mintzberg, 1980), which, as indicated by pertinent prior literature, has never been used in this type of study context.

The outcomes of the present study could serve as a valuable augmentation to the leadership literature in medical education, representing a notable contribution to the research field. Given the historical lack of a conceptual leadership framework in physician leadership education (Matsas *et al.*, 2022; Sultan *et al.*, 2019), Mintzberg’s framework could offer a succinct supplementary tool for leadership education within medical curricula, thereby making a practical contribution to the field.

## Strengths, limitations and, future research

The key strengths of the study lie in the high response rate of 68% (110 out of 162), its methodologically rigorous approach, the use of several forms of triangulation, and the inclusion of diverse research methods to ensure validity and reliability (Eriksson and Kovalainen, 2008). However, the study also has limitations, including the limited sample size and the focus exclusively on final-year medical students from a single university. This study utilized Mintzberg’s framework as a foundation; however, further validation of its application within medical education is necessary to enhance its contextual relevance. Although the instrument was designed to ensure content validity and demonstrated satisfactory reliability, the absence of extensive validation specifically within medical education settings represents a limitation. Future research could address this limitation by conducting additional validation of Mintzberg’s framework in healthcare and medical education environments. Additionally, exploring larger sample sizes and diverse leadership models could deepen our understanding. Comprehensive surveys probing the relationship between management and leadership roles and success could yield richer insights. Comparative studies across universities and countries could also shed light on variations in perceptions and practices.

## Supplementary material

**Table A1:** Cronbach’s alpha reliability analysis: Importance of managerial roles in physician leaders’ work

Subrole	Alpha
Figurehead	0.7654
Leader	0.769
Liaison	0.7609
Monitor	0.7498
Disseminator	0.7414
Spokesman	0.7499
Entrepreneur	0.7561
Disturbance handler	0.7382
Resource allocator	0.7629
Negotiator	0.7402
Cronbach’s alpha reliability analysis	0.7728

**Note(s):** Cronbach’s alpha was calculated for exploratory purposes to evaluate internal consistency across the items representing Mintzberg’s managerial roles (α = 0.77). However, as the roles are conceptually distinct and intended to measure separate constructs, the alpha value should be interpreted cautiously and not as an indicator of the reliability of a single underlying construct

**Source(s):** Authors’ own work
